# Preoperative Serum Thyroglobulin and Its Correlation with the Burden and Extent of Differentiated Thyroid Cancer

**DOI:** 10.3390/cancers12030625

**Published:** 2020-03-08

**Authors:** Hosu Kim, So Young Park, Jun-Ho Choe, Jee Soo Kim, Soo Yeon Hahn, Sun Wook Kim, Jae Hoon Chung, Jaehoon Jung, Tae Hyuk Kim

**Affiliations:** 1 Division of Endocrinology, Department of Medicine, Gyeongsang National University Changwon Hospital, Gyeongsang National University College of Medicine, Changwon 51472, Korea; narulake@naver.com; 2 Division of Endocrinology & Metabolism, Department of Medicine, Thyroid Center, Samsung Medical Center, Sungkyunkwan University School of Medicine, Seoul 06351, Korea; psyou0623@naver.com (S.Y.P.); swkimmd@skku.edu (S.W.K.); jaeh.chung@samsung.com (J.H.C.); 3 Division of Breast and Endocrine Surgery, Department of Surgery, Samsung Medical Center, Sungkyunkwan University School of Medicine, Seoul 06351, Korea; junho.choe@samsung.com (J.-H.C.); jskim0126@skku.edu (J.S.K.); 4 Department of Radiology, Samsung Medical Center, Sungkyunkwan University School of Medicine, Seoul 06351, Korea; sy.hahn@samsung.com

**Keywords:** thyroid cancer, thyroglobulin, lymph nodes, metastasis, clinical decision making

## Abstract

Lymph node metastasis (LNM) in differentiated thyroid cancer (DTC) is usually detected with preoperative ultrasonography; however, this has limited sensitivity for small metastases, and there is currently no predictive biomarker that can help to inform the extent of surgery required. We evaluated whether preoperative serum thyroglobulin levels can predict tumor burden and extent. We retrospectively reviewed the clinical records of 4029 DTC cases diagnosed and treated at a Samsung Medical Center between 1994 and 2016. We reviewed primary tumor size, number and location of LNM, and presence of distant metastases to reveal relationships between tumor burden and extent and preoperative serum thyroglobulin levels. We found a linear association between increasing preoperative thyroglobulin levels, the size of the primary tumor, and the number of LNM (*r* = 0.34, *p* < 0.001, *r* = 0.20, *p* < 0.001, respectively). Tumor extent also increased with each decile of increasing preoperative thyroglobulin level (r = 0.18, *p* < 0.001). Preoperative thyroglobulin levels of 13.15 ng/mL, 30.05 ng/mL, and 62.9 ng/mL were associated with the presence of ipsilateral lateral LNM, contralateral lateral LNM, and distant metastasis, respectively. Our results suggest that preoperative measurement of serum thyroglobulin may help to predict LNM and help to tailor surgery.

## 1. Introduction

Over the last several decades, the incidence of differentiated thyroid cancer (DTC) has been increasing worldwide. In Korea, DTC affects approximately 43.3 per 100,000 individuals in 2014 [1]. Metastasis to the lymph nodes (LNM) is common in DTC, particularly in a subtype of the disease called papillary thyroid carcinoma (PTC) which makes up approximately more than 90% of thyroid cancer [2]. In a study analyzing US data from the Surveillance, Epidemiology, and End Results (SEER) cancer registry, metastasis to nodes in the central and lateral neck has been reported to occur in about 57% and 34% of DTC cases [3,4]. Therefore, central neck lymph node dissection and lateral lymph node neck dissection are often performed depending on the extent of DTC [4]. Local metastasis of DTC to the lymph nodes and the potential for further distant metastasis increases the risk of recurrence of disease and is associated with a poorer prognosis. Therefore, thorough removal of metastatic lymph nodes is essential for effective treatment of DTC [5,6].

The conventional method for detecting metastasis of DTC to the lymph nodes is high-resolution ultrasonography. However, approximately one-third of patients with PTC may have LNM that is missed by preoperative ultrasonography [7]. Furthermore, the accuracy of ultrasonography is highly dependent on radiologist experience [8] which varies considerably among hospitals. False negative results of ultrasonography provide a misleading prognosis of DTC which may lead to conservative treatment that increases the chance of recurrence and disease-specific mortality. We propose that other indicators that can predict the burden and extent of DTC are needed.

In medullary thyroid cancer (MTC), preoperative serum calcitonin levels have been shown to predict the burden and extent of tumor and support decision-making regarding the extent of surgery required. Even when ultrasonography detects no LNM, high levels of serum calcitonin can predict the presence of LNM in MTC [9].

Our own research recently showed that preoperative serum thyroglobulin levels can predict distant metastasis of DTC [10]. We therefore hypothesized that preoperative serum thyroglobulin levels may also be related to tumor burden (primary tumor size, number of LNM) and extent of LNM (including distant metastasis). If so, preoperative thyroglobulin levels may be a useful clinical biomarker to help to determine the optimal surgical extent for DTC resection. Here, we retrospectively reviewed primary tumor size, number and location of LNM, and distant metastases of 4029 DTC cases to determine whether preoperative serum thyroglobulin levels are associated with burden and extent of DTC.

## 2. Results

### 2.1. Baseline Patient Population Characteristics

There were 4738 DTC patients diagnosed at Samsung Medical Center between 1994 and 2016 in whom preoperative serum thyroglobulin was measured. Of these patients, 677 were excluded because a neck resection was not performed, and tumor extent could not be measured. Twenty-eight patients were excluded because of a goiter which, in itself, may affect serum thyroglobulin levels. Four patients who were positive for anti-thyroglobulin antibodies (>100 IU/mL) were also excluded.

We evaluated records from a total of 4029 DTC patients with a median follow-up duration of 6.3 years. Baseline characteristics of the patients are shown in Table 1. The mean age was 46 years, and 77.8% were women. All 4029 patients received a total thyroidectomy with neck lymph node dissection. The lateral neck compartments were dissected systematically in 607 patients (15.1%). The mean primary tumor size was 1.1 cm (range: 0.1–9.0 cm). Metastasis to the lymph nodes was present in 1982 (49.2%) patients. The median number of LNM was three (range: 1 to 49). Among patients with LNM, 1884 (46.8%) patients had central LNM, and 460 (11.4%) patients had lateral LNM. Of the patients with lateral LNM, 348 (8.6%) patients had ipsilateral lateral LNM, and 112 (2.8%) patients had contralateral lateral LNM. Forty-four (1.1%) patients were diagnosed with distant metastasis. One hundred and thirty-eight (3.4%) patients received a secondary operation following recurrence of DTC. The five-year recurrence-free survival rate was 96.2%. Fifteen (0.4%) patients died during follow-up and, of these, eight (0.2%) patients died due to DTC. The five-year cancer-specific survival rate was 99.8%.

The median preoperative thyroglobulin level was 9.9 ng/mL (range 0.1–42,680 ng/mL). Because preoperative thyroglobulin levels did not increase linearly, we grouped patients into equal size deciles by their preoperative serum thyroglobulin levels with each decile representing a range of serum thyroglobulin levels.

To find the factors which correlated with preoperative serum thyroglobulin levels, we analyzed multiple linear regression with clinical variables. When adjusted for other variables, the factors significantly associated with preoperative serum thyroglobulin levels were age, sex (female), tumor histology (FTC), primary tumor size, number of LNM, positive resection margin, and presence of distant metastasis (Table 2).

### 2.2. Tumor Burden and Recurrence According to Preoperative Thyroglobulin Levels

We next analyzed the relationship between preoperative thyroglobulin levels and primary tumor size, number of LNM and recurrence (Table 3). We found that, with deciles of increasing preoperative thyroglobulin level, there was a statistically significant increase in the mean size of the primary tumor (*r* = 0.34, *p* < 0.001) and in the mean number of LNM (*r* = 0.20, *p* < 0.001 for number of LNM). Recurrence also increased with increasing preoperative serum thyroglobulin levels (*r* = 0.13, *p* < 0.001). The number of disease-specific deaths was also associated with deciles of preoperative thyroglobulin levels (*r* = 0.05, *p* = 0.001), except in one patient whose preoperative thyroglobulin level was 2.3 ng/mL. After adjusting for other factors (i.e., age, sex, tumor histology, and resection margins), there was also statistical significance between tumor burden and serum thyroglobulin levels (*β* ± SE = 0.10 ± 0.01, *p* < 0.001 for tumor size and *β* ± SE = 0.36 ± 0.02, *p* < 0.001 for number of LNM).

**Figure 1:**
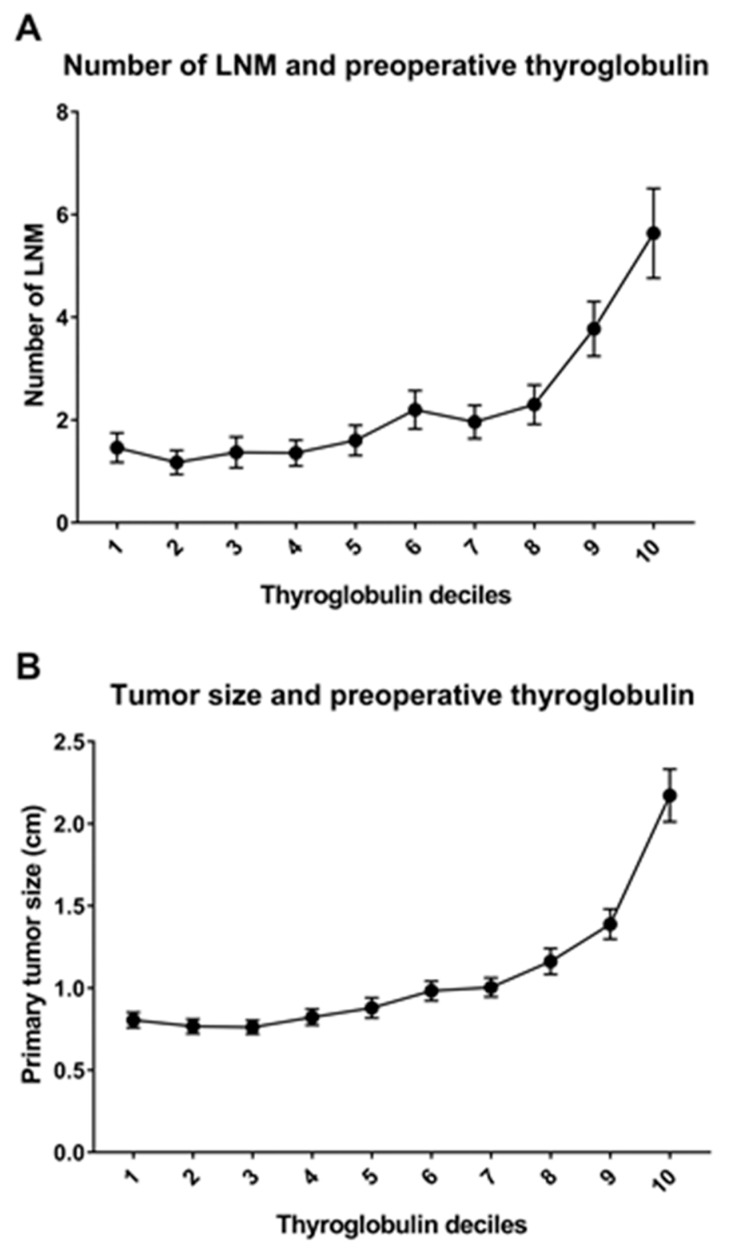
Correlation between tumor burden and preoperative thyroglobulin levels. There is a linear relationship between preoperative serum thyroglobulin and (**A**) the number of LNM and (**B**) the size of the primary tumor. In patients in the eighth and ninth decile of preoperative thyroglobulin levels, tumor size and the number of LNM increase sharply. Thyroglobulin deciles: the deciles of patients with different levels of preoperative thyroglobulin levels (levels increase from deciles 1 to 10).

Moreover, when the mean number of LNM and mean tumor size is plotted against each decile of increasing thyroglobulin (Figure 1), a sharp increase can be seen in both tumor size and LNM when the preoperative thyroglobulin level exceeds 17.9 ng/mL (the lower level for the eighth decile) and 26.8 ng/mL (the lower level for the ninth decile), respectively (Figure 1). Subgroup analyses for papillary thyroid carcinoma and follicular thyroid carcinoma are provided as supplementary data (Supplementary Materials Tables S1 and S2; Figures S1 and S2).

### 2.3. Tumor Extent According to Preoperative Thyroglobulin Levels

**Figure 2:**
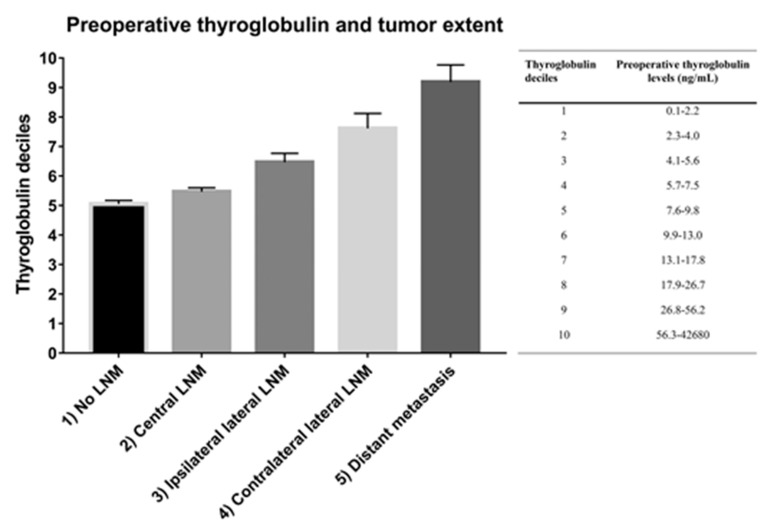
Correlation between tumor extent and preoperative thyroglobulin levels. Tumor extent was classified as (1) no lymph node metastases (LNM), (2) central LNM, (3) ipsilateral lateral LNM, (4) contralateral lateral LNM, and (5) distant metastasis. We found a linear association between the proportion of patients with higher tumor extent and increasing preoperative serum thyroglobulin levels. Thyroglobulin deciles: the deciles of patients with different levels of preoperative thyroglobulin levels (levels increase from 1 to 10).

Among the 1982 patients with LNM, 1512 (37.5%) patients had central LNM only. Of the 437 patients with lateral LNM (no distant metastasis), 334 (8.3%) had ipsilateral and 103 (2.6%) had contralateral lateral LNM. Forty-four (1.1%) patients had distant metastasis regardless of LNM. Figure 2 shows the relationship between increasing deciles of serum thyroglobulin levels and the extent of disease. As the extent of disease increases from no LNM (1) through to distant metastasis (5) so do the preoperative thyroglobulin levels (*r* = 0.18, *p* < 0.001). Patients in the fifth decile of thyroglobulin levels (i.e., from 7.6–9.8 ng/mL upwards) had ipsilateral lateral LNM, whereas thyroglobulin levels exceeding 13.1 to 17.8 ng/mL were associated with contralateral lateral LNM (Table 4). Distant metastasis was observed at preoperative serum thyroglobulin levels 2.3 ng/mL or higher, and a sharp increase in distant metastasis was seen in patients in the tenth decile (thyroglobulin range 56.3 to 42,680 ng/mL). After adjusting for other factors, tumor extent and preoperative thyroglobulin levels still had significant association (*β* ± SE = 0.06 ± 0.01, *p* < 0.001). Subgroup analyses for papillary thyroid carcinoma and follicular thyroid carcinoma are provided as supplementary data (Supplementary Materials Tables S3 and S4; Figures S3 and S4).

In the ROC analysis, the preoperative serum thyroglobulin cutoff level for predicting ipsilateral lateral LNM was 13.15 ng/mL (sensitivity 57.0%, specificity 60.6%) and for predicting contralateral lateral LNM was 30.05 ng/mL (sensitivity 56.6%, specificity 81.4%). As with our previous study, initial distant metastasis was associated with a preoperative serum thyroglobulin level greater than 62.9 ng/mL (sensitivity 85.0%, specificity 90.6%) [10]. Taken together, these results suggest that preoperative thyroglobulin level is significantly associated with tumor burden and tumor extent.

## 3. Discussion

In this study, we examined the association between preoperative serum thyroglobulin levels, tumor burden, and extent of DTC. As tumor burden (i.e., primary tumor size and the number of LNM) and tumor extent (i.e., presence of lateral LNM and distant metastasis) increased, we found that preoperative serum thyroglobulin increased. Specifically, a preoperative thyroglobulin level above 13.15 ng/mL could predict the presence of an ipsilateral lateral LNM, above 30.05 ng/mL could predict the presence of contralateral lateral LNM, and above 62.9 ng/mL could predict the presence of distant metastasis.

Our findings are significant, because the incidence of thyroid cancer has increased substantially worldwide [11,12,13], a trend that is attributed to the overdiagnosis of small PTC. As a result, the current treatment guideline of DTC has become more conservative [14,15], and lobectomy has been performed more frequently in the treatment of low-risk DTC [4,16]. However, even if a DTC is completely resected, it may recur later, resulting in a poorer prognosis and requiring additional operations that can adversely impact patient health-related quality of life [17]. There is a significant unmet need to be able to more accurately predict, in the preoperative stage, the full tumor extent to help guide surgical planning and balance the need to reduce recurrence and ensure optimal recovery from treatment. Our results suggest that preoperative serum thyroglobulin levels may provide additional information to ultrasonography in the preoperative setting rather than definite diagnosis of metastasis. For example, in a patient who has high preoperative serum thyroglobulin levels during the preoperative laboratory work-up for a lobectomy for low-risk DTC, surgeons should order cross-sectional imaging and consider a total thyroidectomy with or without additional neck dissection. Furthermore, if the preoperative thyroglobulin level is unequivocally high, clinicians should consider advanced whole-body imaging studies in pursuit of distant metastases.

Of note, even in a small-sized primary DTC of less than 1 cm diameter, LNM was frequently detected [18]. In our study, among patients with a primary tumor diameter less than 1 cm, 992 (40.6%) had LNM and the smallest primary tumor diameter in patients with LNM was 0.1 cm. In addition, 147 (32%) patients with lateral LNM had a primary tumor of less than 1 cm and the smallest primary tumor diameter that also presented with lateral LNM was 0.2 cm. If these patients were treated solely with lobectomy based on their small tumor size, there would be a high likelihood of recurrence after an incomplete initial surgery. Therefore, primary tumor size alone does not reflect tumor extent and cannot accurately guide surgical treatment planning. In such cases, preoperative thyroglobulin could better inform clinical decision making, providing information on the true tumor extent.

The inclusion of a thyroglobulin blood test at the preoperative stage may be particularly relevant in low-resource settings where there is a paucity of skilled radiologists to facilitate accurate preoperative clinical staging.

Although preoperative thyroglobulin was helpful for predicting tumor burden and extent in DTC, it is not as specific as calcitonin is for predicting MTC [9]. This difference can be mostly attributed to the fact that thyroglobulin is also produced in normal thyroid tissue before thyroidectomy, while calcitonin is mainly produced in MTC cells or parafollicular (C) cells [19]. One recent study has shown that preoperative thyroglobulin has negative results for predicting metastasis in DTC [20]. For the same reason, it is not possible to determine the extent of surgery based on preoperative thyroglobulin results alone. However, with the recent trend of choosing lobectomy as a preferred surgical option, preoperative thyroglobulin levels can provide ancillary information about tumor extent before surgery, help to determine the extent of surgery, and flag cases for which a lobectomy may not be sufficient.

There were some limitations to this study. There is a bias inherent to the retrospective study setting and single center design. Because patients were recruited from a single teaching hospital, they were prone to a selection bias. External validation using a multicenter prospective study is needed in the future.

## 4. Materials and Methods

We used the institutional thyroid cancer database and retrospectively reviewed the clinical records of patients who were diagnosed and treated with DTC at Samsung Medical Center from 1994 to 2016. This study was approved by the institutional review board (IRB) of Samsung Medical Center (IRB File No. 2017-02-056) and performed in accordance with relevant guidelines and regulations. Informed consent was waived due to the retrospective design and the anonymization of all patient data.

Pathologic data on primary tumor size (diameter; cm) and the total number of LNM from patients’ anonymized medical records were used to determine tumor burden. To evaluate tumor extent, the location of LNM (i.e., central, ipsilateral lateral or contralateral lateral) and the presence of distant metastasis were ascertained from the clinical data for each patient. Distant metastasis was defined as a suspicious metastatic lesion detected before or within 6 months after initial surgery. Distant metastasis was found by pathological confirmation or imaging study such as computed tomography (CT), magnetic resonance imaging, whole-body scan (WBS), or positron emission tomography scans. Tumor extent was classified as (1) no metastasis; (2) presence of central LNM with no lateral LNM and no distant metastasis; (3) presence of ipsilateral lateral LNM with no contralateral lateral LNM and no distant metastasis, regardless of central LNM status; (4) presence of contralateral lateral LNM with no distant metastasis, regardless of central and ipsilateral lateral LNM; and (5) presence of distant metastasis, regardless of LNM. Central LNM did not distinguish between ipsilateral and contralateral LNM. Other primary tumor histology parameters, such as lymphatic invasion, blood vessel invasion, extrathyroidal extension (ETE), and resection margins, were also reviewed from clinical data.

Patients checked serum thyroglobulin levels from blood sampling preoperatively. Serum thyroglobulin was measured using immune-radiometric assays with a BRAHMS Thyroglobulin plus RIA assay (BRAHMS, Henningsdoft, Germany). The functional sensitivity of the Thyroglobulin plus RIA assay was 0.2 ng/mL, and the analytical sensitivity was 0.008 ng/mL. The intraassay coefficient of variation (CV) of the assay was 3.4%, and the interassay CV was 4.9% [21].

### Statistical Analysis

For statistical analysis, continuous data are expressed in the format of mean ± standard deviation. Categorical data are expressed as percent values or absolute numbers. Preoperative serum thyroglobulin is presented as the median (range) in ng/mL. Pearson’s correlation analysis was used for comparison of continuous data and is represented by the correlation coefficient, *r*. Multivariate linear regression was used to analyze factors that affected preoperative thyroglobulin level elevation and is represented by the regression coefficient, β. The enter method was used to adjust other variables. Receiver operative characteristic (ROC) curve analysis was used to determine the threshold level for preoperative serum thyroglobulin to distinguish tumor extent. Sensitivity and specificity were calculated using an established method. A *p*-value of <0.05 was considered to be significant. Statistical analysis was performed using the Statistical Package for the Social Sciences software version 23 (IBM Corp., Armonk, NY, USA).

## 5. Conclusions

In conclusion, preoperative thyroglobulin can predict the LNM and overall extent of DTC. The results obtained here suggest that preoperative measurement of thyroglobulin may help to optimize surgical planning in newly diagnosed DTC patients, reducing the number of reoperations and improving prognosis.

## Figures and Tables

**Table 1:** Baseline characteristics of patients included in the analysis.

Characteristics	Data
Age at diagnosis (Years)	46.43 ± 11.34
Sex	
Female	3135 (77.8%)
Male	894 (22.2%)
Tumor histology	
Papillary thyroid carcinoma	3979 (98.8%)
Follicular thyroid carcinoma	50 (1.2%)
Tumor size (diameter; cm)	1.06 ± 0.84
Extrathyroidal extension	
None	1685 (41.8%)
Microscopic	1773 (44.0%)
Gross	571 (14.2%)
Positive resection margin	
No	3850 (95.6%)
Yes	179 (4.4%)
Positive lymphatic invasion	
No	3973 (98.6%)
Yes	56 (1.4%)
Positive vascular invasion	
No	3972 (98.6%)
Yes	57 (1.4%)
Extent of LNM	
Central LNM	1884 (46.8%)
Ipsilateral lateral LNM	348 (8.6%)
Contralateral lateral LNM	112 (2.8%)
Distant metastasis	
No	3985 (98.9%)
Yes	44 (1.1%)
Overall deaths	15 (0.4%)
Disease-specific deaths	8 (0.2%)

Continuous data are reported as the mean ± SD; categorical data are reported as the absolute numbers (percentage values). LNMs—lymph node metastases.

**Table 2:** Multivariate linear regression analysis results showing factors associated with preoperative serum thyroglobulin deciles.

Characteristics	Unadjusted		Adjusted	
	*β* ± SE	*p*-Value	*β* ± SE	*p*-Value
Age at diagnosis (years)	−0.002 ± 0.004	0.628	0.010 ± 0.004	0.0009
Sex (female)	0.291 ±0.108	0.007	0.532 ± 0.101	<0.001
Tumor histology (FTC)	2.021 ± 0.406	<0.001	0.828 ± 0.400	0.039
Tumor size	0.830 ± 0.041	<0.001	1.078 ± 0.056	<0.001
Positive lymphatic invasion	1.678 ± 0.384	<0.001	0.315 ± 0.360	0.382
Positive blood vessel invasion	1.800 ± 0.381	<0.001	−0.156 ± 0.372	0.674
Number of LNM	0.160 ± 0.010	<0.001	0.091 ± 0.011	<0.001
Positive ETE	0.595 ± 0.064	<0.001	−0.009 ± 0.065	0.889
Positive resection margin	1.534 ± 0.217	<0.001	0.662 ± 0.206	0.001
Presence of distant metastasis	4.066 ± 0.439	<0.001	0.577 ± 0.424	0.174

Thyroglobulin deciles: the deciles of patients with different levels of preoperative thyroglobulin levels (levels increase from 1 to 10). SE—standard error, FTC—follicular thyroid carcinoma, LNM—lymph node metastases, ETE—extrathyroidal extension.

**Table 3:** Tumor burden and cancer prognosis by preoperative serum thyroglobulin levels.

Thyroglobulin Deciles	Preoperative Thyroglobulin Levels (ng/mL)	*n*	Primary Tumor Diameter (cm)		Number of LNM		Recurrence		DSD	
			Mean	95% CI	Mean	95% CI	*n*	%	*n*	%
1	0.1–2.2	387	0.81	0.76; 0.86	1.42	1.13; 1.72	5	1.3	0	0
2	2.3–4.0	399	0.76	0.71; 0.80	1.16	0.93; 1.39	6	1.5	1	0.3
3	4.1–5.6	407	0.77	0.73; 0.81	1.33	1.03; 1.62	6	1.5	0	0
4	5.7–7.5	406	0.82	0.77; 0.87	1.34	1.08; 1.60	7	1.7	0	0
5	7.6–9.8	404	0.86	0.80; 0.92	1.67	1.37; 1.97	8	2	0	0
6	9.9–13.0	408	0.98	0.92; 1.04	2.02	1.66; 2.38	12	2.9	0	0
7	13.1–17.8	405	1	0.94; 1.06	2.06	1.72; 2.39	13	3.2	0	0
8	17.9–26.7	404	1.16	1.08; 1.24	2.25	1.88; 2.62	16	4	0	0
9	26.8–56.2	404	1.31	1.22; 1.40	3.32	2.83; 3.81	21	5.2	1	0.2
10	56.3–42680	405	2.07	1.93; 2.21	5.48	4.71; 6.25	44	10.9	6	1.5
Total		4029	1.06	1.03; 1.08	2.21	2.01; 2.34	138	3.4	8	0.2

Thyroglobulin deciles: the deciles of patients with different levels of preoperative thyroglobulin levels (levels increase from 1 to 10). LNM—lymph node metastases, DSD—disease-specific death, CI—confidence interval.

**Table 4:** Tumor extent by preoperative serum thyroglobulin levels.

Thyroglobulin Deciles	Preoperative Thyroglobulin Levels (ng/mL)	*n*	Central LNM		Ipsilateral Lateral LNM		Contralateral Lateral LNM		Distant Metastasis	
			*n*	%	*n*	%	*n*	%	*n*	%
1	0.1–2.2	387	135	34.9	21	5.4	5	1.3	0	0.0
2	2.3–4.0	399	145	36.3	22	5.5	1	0.3	1	0.3
3	4.1–5.6	407	149	36.6	22	5.4	5	1.2	0	0.0
4	5.7–7.5	406	156	38.4	16	3.9	4	1.0	1	0.2
5	7.6–9.8	404	160	39.6	33	8.2	4	1.0	2	0.5
6	9.9–13.0	408	165	40.4	31	7.6	5	1.2	0	0.0
7	13.1–17.8	405	144	35.6	36	8.9	13	3.2	1	0.2
8	17.9–26.7	404	157	38.9	41	10.1	11	2.7	1	0.2
9	26.8–56.2	404	158	39.1	53	13.1	20	5.0	3	0.7
10	56.3–42680	405	143	35.3	59	14.6	35	8.6	35	8.6
Total		4029	1512	37.5	334	8.3	103	2.6	44	1.1

Thyroglobulin deciles: the deciles of patients with different levels of preoperative thyroglobulin levels (levels increase from 1 to 10). LNM—lymph node metastases.

## Supplementary Materials

The following are available online at https://www.mdpi.com/2072-6694/12/3/625/s1, Figure S1: Correlation between tumor burden and preoperative thyroglobulin levels in patients with papillary thyroid carcinoma, Figure S2: Correlation between tumor burden and preoperative thyroglobulin levels in patients with follicular thyroid carcinoma, Figure S3: Correlation between tumor extent and preoperative thyroglobulin levels in patients with papillary thyroid carcinoma, Figure S4: Correlation between tumor extent and preoperative thyroglobulin levels in patients with follicular thyroid carcinoma, Table S1: Tumor burden and cancer prognosis by preoperative serum thyroglobulin levels in patients with papillary thyroid carcinoma, Table S2: Tumor burden and cancer prognosis by preoperative serum thyroglobulin levels in patients with follicular thyroid carcinoma, Table S3: Tumor extent by preoperative serum thyroglobulin levels in patients with papillary thyroid carcinoma, Table S4. Tumor extent by preoperative serum thyroglobulin levels in patients with follicular thyroid carcinoma.
