# Supplementary material for: Preoperative Serum Thyroglobulin and Its Correlation with the Burden and Extent of Differentiated Thyroid Cancer
Source: Cancers (Basel). 2020 Mar 8;12(3):625. doi: 10.3390/cancers12030625 (PMC7139752; doi:10.3390/cancers12030625)
Supplement: Supplementary file 1 [file cancers-12-00625-s001.pdf]

Supplementary Materials: Preoperative Serum Thyroglobulin and Its Correlation with the Burden and Extent of Differentiated Thyroid Cancer

Hosu Kim, So Young Park, Jun-Ho Choe, Jee Soo Kim, Soo Yeon Hahn, Sun Wook Kim, Jae Hoon Chung, Jaehoon Jung and Tae Hyuk Kim

Table S1. Tumor burden and cancer prognosis by preoperative serum thyroglobulin levels in patients with papillary thyroid carcinoma.

Thyroglobulin deciles	Preoperative Thyroglobulin Levels (ng/mL)	*n*	Primary tumor Diameter (cm)		Number of LNM		Recurrence		DSD	
			mean	95% CI	mean	95% CI	*n*	%	*n*	%
1	0.1–2.2	386	0.81	0.76;0.86	1.44	1.14;1.73	5	1.3	0	0
2	2.3–4.0	395	0.76	0.71;0.80	1.16	0.93;1.40	6	1.5	1	0.3
3	4.1–5.6	403	0.77	0.73;0.81	1.33	1.03;1.63	6	1.5	0	0
4	5.7–7.5	401	0.82	0.77;0.87	1.34	1.08;1.61	7	1.7	0	0
5	7.6–9.8	404	0.86	0.80;0.92	1.67	1.37;1.96	8	2	0	0
6	9.9–13.0	407	0.98	0.92;1.04	2.02	1.66;2.38	12	2.9	0	0
7	13.1–17.8	402	1	0.94;1.06	2.06	1.73;2.40	13	3.2	0	0
8	17.9–26.7	401	1.16	1.08;1.24	2.27	1.89;2.64	16	4	0	0
9	26.8–56.2	398	1.31	1.22;1.40	3.37	2.83;3.87	21	5.3	1	0.8
10	56.3–42680	382	2	1.86;2.14	5.74	4.93;6.54	43	11.3	3	0.8
total		3979	1.04	1.02;1.07	2.22	2.09;2.36	137	3.4	5	0.1

Thyroglobulin deciles: the deciles of patients with different levels of preoperative thyroglobulin levels (levels increase from 1 to 10). LNM—lymph node metastases, DSD—disease-specific death, CI—confidence interval.

Table S2. Tumor burden and cancer prognosis by preoperative serum thyroglobulin levels in patients with follicular thyroid carcinoma.

Thyroglobulin Deciles	Preoperative Thyroglobulin Levels (ng/mL)	*n*	Primary tumor Diameter (cm)		Number of LNM		Recurrence		DSD	
			mean	95% CI	mean	95% CI	*n*	%	*n*	%
1	0.1–2.2	1	-	-	-	-	0	0	0	0
2	2.3–4.0	4	0.88	0.48;1.27	0.75	−0.05;1.55	0	0	0	0
3	4.1–5.6	4	1.03	0.22;1.83	0.75	−0.77;2.27	0	0	0	0
4	5.7–7.5	5	0.88	−0.25;2.01	1.20	−1.49;3.89	0	0	0	0
5	7.6–9.8	0	-	-	-	-	0	0	0	0
6	9.9–13.0	1	-	-	-	-	0	0	0	0
7	13.1–17.8	3	1.10	−0.84;3.04	1.33	−4.40;7.07	0	0	0	0
8	17.9–26.7	3	0.87	−0.17;1.90	-	-	0	0	0	0
9	26.8–56.2	6	2.15	1.08;3.22	0.17	−0.26;0.60	0	0	0	0
10	56.3–42680	23	3.27	2.69;3.86	1.26	−0.23;2.75	1	4.3	3	13.0
total		50	2.14	1.70;2.57	0.92	0.21;1.63	1	2.0	3	6.0

Thyroglobulin deciles: the deciles of patients with different levels of preoperative thyroglobulin levels (levels increase from 1 to 10). LNM—lymph node metastases, DSD—disease-specific death, CI—confidence interval.

Table S3. Tumor extent by preoperative serum thyroglobulin levels in patients with papillary thyroid carcinoma.

Thyroglobulin Deciles	Preoperative Thyroglobulin Levels (ng/mL)	*n*	Central LNM		Ipsilateral lateral LNM		Contralateral Lateral LNM		Distant Metastasis	
			n	%	*n*	%	*n*	%	*n*	%
1	0.1–2.2	386	135	35.0	21	5.4	5	1.3	0	0.0
2	2.3–4.0	395	142	35.9	22	5.6	1	0.3	1	0.3
3	4.1–5.6	403	148	36.7	21	5.2	5	1.2	0	0.0
4	5.7–7.5	401	154	38.4	16	4.0	4	1.0	1	0.2
5	7.6–9.8	404	160	39.6	33	8.2	4	1.0	2	0.5
6	9.9–13.0	407	165	40.5	31	7.6	5	1.2	0	0.0
7	13.1–17.8	402	142	35.3	36	9.0	13	3.2	1	0.2
8	17.9–26.7	401	157	39.2	41	10.2	11	2.7	1	0.2
9	26.8–56.2	398	157	39.4	53	13.3	20	5.0	3	0.8
10	56.3–42680	382	143	37.4	58	15.2	33	8.6	25	6.5
	total	3979	1503	37.8	332	8.3	101	2.5	34	0.9

Thyroglobulin deciles: the deciles of patients with different levels of preoperative thyroglobulin levels (levels increase from 1 to 10). LNM—lymph node metastases.

Table S4. Tumor extent by preoperative serum thyroglobulin levels in patients with follicular thyroid carcinoma.

Thyroglobulin Deciles	Preoperative Thyroglobulin Levels (ng/mL)	*n*	Central LNM		Ipsilateral Lateral LNM		Contralateral Lateral LNM		Distant Metastasis	
			n	%	*n*	%	*n*	%	*n*	%
1	0.1–2.2	1	0	0	0	0	0	0	0	0
2	2.3–4.0	4	3	75.0	0	0	0	0	0	0
3	4.1–5.6	4	1	25.0	1	25.0	0	0	0	0
4	5.7–7.5	5	2	40.0	0	0	0	0	0	0
5	7.6–9.8	0	0	0	0	0	0	0	0	0
6	9.9–13.0	1	0	0	0	0	0	0	0	0
7	13.1–17.8	3	2	66.7	0	0	0	0	0	0
8	17.9–26.7	3	0	0	0	0	0	0	0	0
9	26.8–56.2	6	1	16.7	0	0	0	0	0	0
10	56.3–42680	23	0	0	1	4.3	2	8.7	10	43.5
	total	50	9	18.0	2	4.0	2	4.0	10	20.0

Thyroglobulin deciles: the deciles of patients with different levels of preoperative thyroglobulin levels (levels increase from 1 to 10). LNM—lymph node metastases.

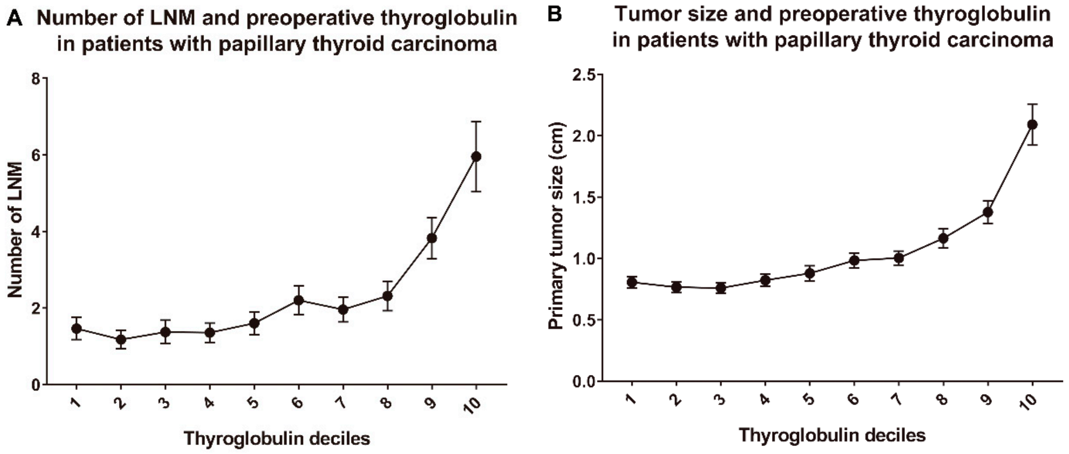

Figure S1. Correlation between tumor burden and preoperative thyroglobulin levels in patients with papillary thyroid carcinoma. (**a**) the number of LNM and (**b**) the size of the primary tumor.

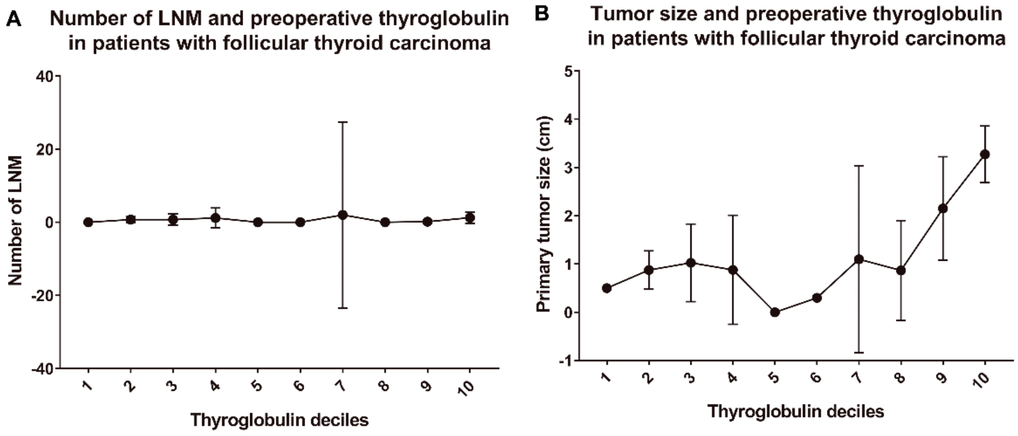

Figure S2. Correlation between tumor burden and preoperative thyroglobulin levels in patients with follicular thyroid carcinoma. (**a**) the number of LNM and (**b**) the size of the primary tumor.

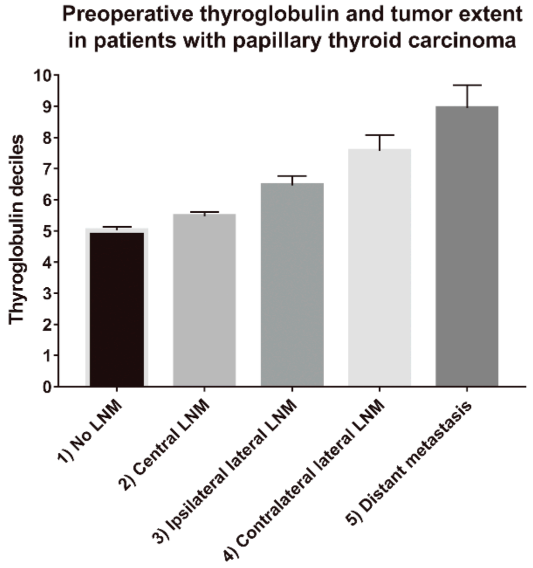

Figure S3. Correlation between tumor extent and preoperative thyroglobulin levels in patients with papillary thyroid carcinoma.

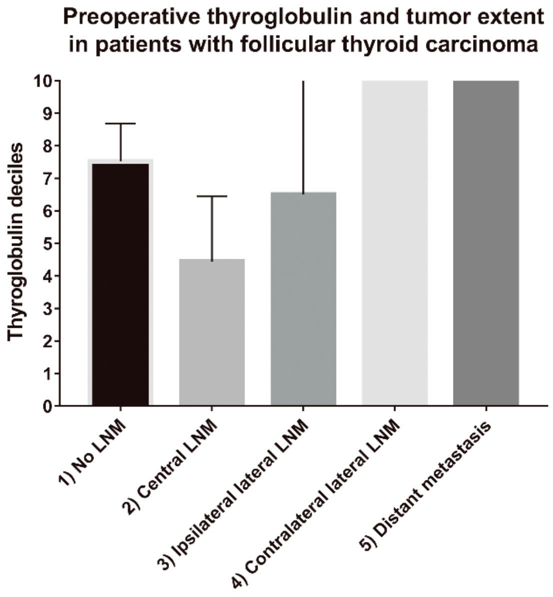

Figure S4. Correlation between tumor extent and preoperative thyroglobulin levels in patients with follicular thyroid carcinoma.

© 2020 by the authors. Licensee MDPI, Basel, Switzerland. This article is an open access article distributed under the terms and conditions of the Creative Commons Attribution (CC BY) license (http://creativecommons.org/licenses/by/4.0/).
